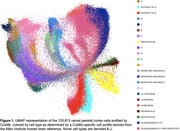# Spatial molecular characterization of early Alzheimer's disease neuropathology in aged Vervet monkeys

**DOI:** 10.1002/alz70855_106882

**Published:** 2025-12-24

**Authors:** Caitlin S Latimer, Miranda E. Orr, Emily E. Killingbeck Schneidereit, William Harrison, Angela M Wilson, Mamatha Damodarasamy, Timothy C. Orr, C. Dirk Keene, Suzanne Craft, Carol A. Shively, Thomas C. Register

**Affiliations:** ^1^ University of Washington, Seattle, WA, USA; ^2^ Wake Forest University School of Medicine, Winston‐Salem, NC, USA; ^3^ Washington University School of Medicine, St. Louis, MO, USA; ^4^ University of Washington, School of Medicine, Seattle, WA, USA; ^5^ Wake Forest Alzheimer's Disease Research Center, Winston‐Salem, NC, USA

## Abstract

**Background:**

Vervets (Chlorocebus aethiops) are non‐human primates in which we previously demonstrated brain amyloid b (Ab) deposition and phosphorylated tau associations with reduced brain volume, glucose metabolism, and gait speed but lacking true neurofibrillary tangles (NFTs). Vervets represent an excellent model of the earliest (pre‐NFT) stages of AD, a period when therapeutic intervention may be efficacious. Molecular analyses of AD in human brain have continued to advance but similar approaches in aged NHPs are lacking. We performed spatially conserved molecular characterization in aged vervet brain, aligned with human analyses, to identify regional and cell type specific changes in the presence of Aβ plaques.

**Method:**

Fixed tissue brain sections from 10 vervets (X=24.3 years, ∼95 human years) were evaluated using the NIA‐Alzheimer's Association Guidelines for the Neuropathologic Assessment of Alzheimer's Disease. Single cell spatial transcriptomics was performed using the NanoString CosMx Spatial Molecular Imager and the human RNA 6K Discovery Panel on fresh frozen parietal cortex sections to identify cell type specific changes in the presence of Aβ plaques, confirmed with immunofluorescent co‐labeling. In a subset of 5 vervets, the NanoString GeoMx Digital Spatial Profiler was used to assess protein content and distribution within and around neurons and amyloid plaques.

**Result:**

Aβ plaques were associated with increased content of Ab‐42, Ab‐42, IBA1, GFAP, APP, and autophagy related proteins. Neurons adjacent to plaques, as compared to those distant to plaques, also showed differential expression of some proteins. The CosMx 6K human discovery panel performed well for characterizing vervet parietal cortex, averaging ∼1100 transcripts per cell across ∼126,000 cells. Using this spatially‐localized transcript expression and the CosMx Allen brain reference, we annotated cell types of interest for AD including neurons, microglia, astrocytes, oligodendrocytes, and endothelial cells, and mapped their distribution (Figure 1).

**Conclusion:**

Opportunities to systematically study early AD in humans are scarce, and late in the disease progression vulnerable brain regions are devastated by pathology. The current results demonstrate the significant potential of leveraging powerful molecular techniques in the vervet model of early AD to probe cell type specific mechanisms driving the earliest changes of AD and thus identify novel therapeutic targets.